# Acute Progressive Spinal Arachnoid Web Without the Scalpel Sign: A Case Report

**DOI:** 10.7759/cureus.78988

**Published:** 2025-02-14

**Authors:** Sho Nakamura, Shinsuke Yoshida, Ikuo Kobayashi, Tsubasa Sonoda, Masahiro Indo

**Affiliations:** 1 Department of Neurosurgery, Higashi-Yamato Hospital, Higashiyamato, JPN; 2 Department of Neurosurgery, Saitama Medical Center, Kawagoe, JPN

**Keywords:** acute onset, scalpel sign, spinal arachnoid web, surgery, syringomyelia

## Abstract

Spinal arachnoid web (SAW) is a chronic disorder characterized by thickened arachnoid tissue obstructing the cerebrospinal fluid flow, leading to spinal cord compression and often associated with syringomyelia. We report an uncommon case of a 77-year-old man with SAW who presented with acute progressive neurological deterioration. Spinal cord deformation and displacement identified on initial imaging prompted the use of advanced imaging techniques, such as heavily T2-weighted magnetic resonance imaging (MRI), which facilitated a timely diagnosis and subsequent surgical intervention. Microsurgical web excision with laminectomy was selected as the surgical treatment. Intraoperative findings were consistent with the typical characteristics of SAW, showing no discrepancies with the preoperative diagnosis. After the operation, the patient displayed considerable neurological recovery, including the resolution of spinal cord distortion and normalization visible on MRI. This case underscores the importance of including SAW in the differential diagnosis of acute progressive thoracic myelopathy and highlights the critical role of advanced imaging and early surgical intervention in achieving favorable clinical outcomes.

## Introduction

Spinal arachnoid web (SAW) is a rare pathological entity that causes spinal cord compression due to the obstruction of the cerebrospinal fluid (CSF) flow by a thickened arachnoid membrane [1]. The lesion is typically located dorsally at the thoracic level of the spinal cord [2] and frequently associated with syringomyelia [3]. Common neurological symptoms of SAW include back pain, myelopathy, motor weakness, sensory deficits, sphincter dysfunction, and radiculopathy [4-6], progressing symptoms slowly and mildly. Consequently, delays in diagnosis and surgical treatment can lead to limited symptom improvement, which poses a significant clinical concern [7]. This case highlights the potential for even subtle findings of SAW on imaging to rapidly worsen neurological symptoms. Early diagnosis and timely surgical intervention are crucial for improving clinical outcomes.

## Case presentation

A 77-year-old man developed sensory disturbances in both lower extremities without pain, beginning two days before admission. These symptoms rapidly progressed to lower extremity weakness, sensory deficits, and voiding dysfunction. His mobility deteriorated significantly, necessitating an emergency ambulance transfer to a nearby hospital specializing in spinal and neurological disorders. Despite a thorough evaluation, no definitive diagnosis was established, and the patient was subsequently referred to our facility for further management. The patient's medical history included lumbar disc herniation surgery at the L4/5 level one year earlier and clipping surgery for aneurysmal subarachnoid hemorrhage 13 years prior. Neurological examination revealed approximately 4/5 strength in all lower extremity muscles on the Manual Muscle Test, diminished proprioception below the T4 dermatome, and reduced vibratory sensation. Bilateral patellar tendon reflex and Achilles tendon reflex were increased. The Babinski reflex was positive bilaterally, and progressive bladder and bowel dysfunction were noted. Anal and bulbocavernosus reflexes were slightly diminished.

**Figure 1:**
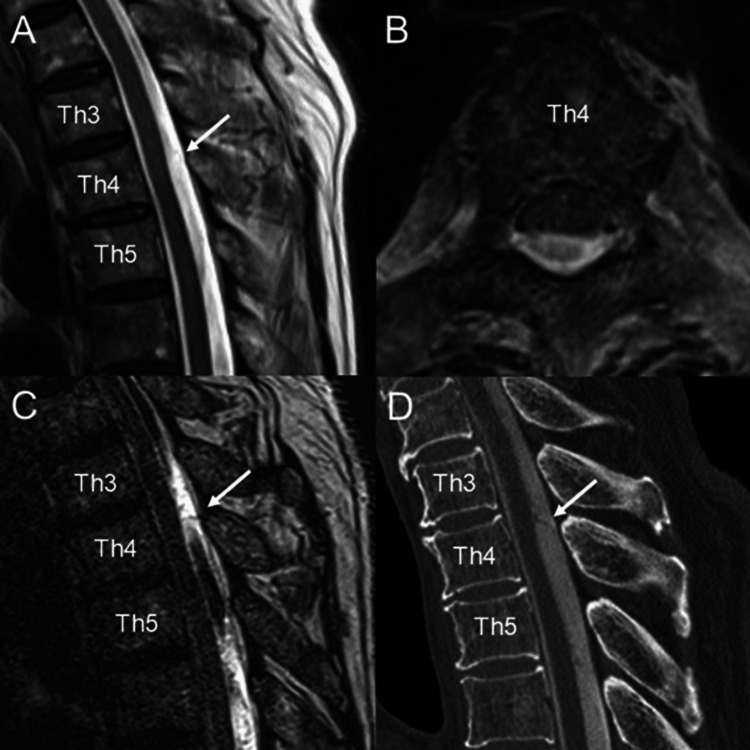
(A, B) Sagittal and axial T2-weighted images of the thoracic spine show mild dorsal compressive deflection of the spinal cord at the T4 level (white arrow). (C, D) A sagittal heavily T2-weighted image (C) and CT myelography (D) of the thoracic spine demonstrate a membrane-like structure dorsal to the spinal cord at the T4 level, suggestive of a SAW (white arrow). CT: computed tomography; SAW: spinal arachnoid web

On imaging findings, the initial conventional magnetic resonance imaging (MRI) demonstrated anterior distortion of the spinal cord at the T4 level in both sagittal and axial views (Figure 1A, 1B). Heavily T2-weighted imaging and computed tomography (CT) myelography provided a clearer depiction of spinal cord compression and a thickened arachnoid membrane at the T4 level (Figure 1C, 1D), suggesting a diagnosis of SAW. The neurological symptoms correlated with the high level of SAW, which was determined to be the underlying cause.

**Figure 2:**
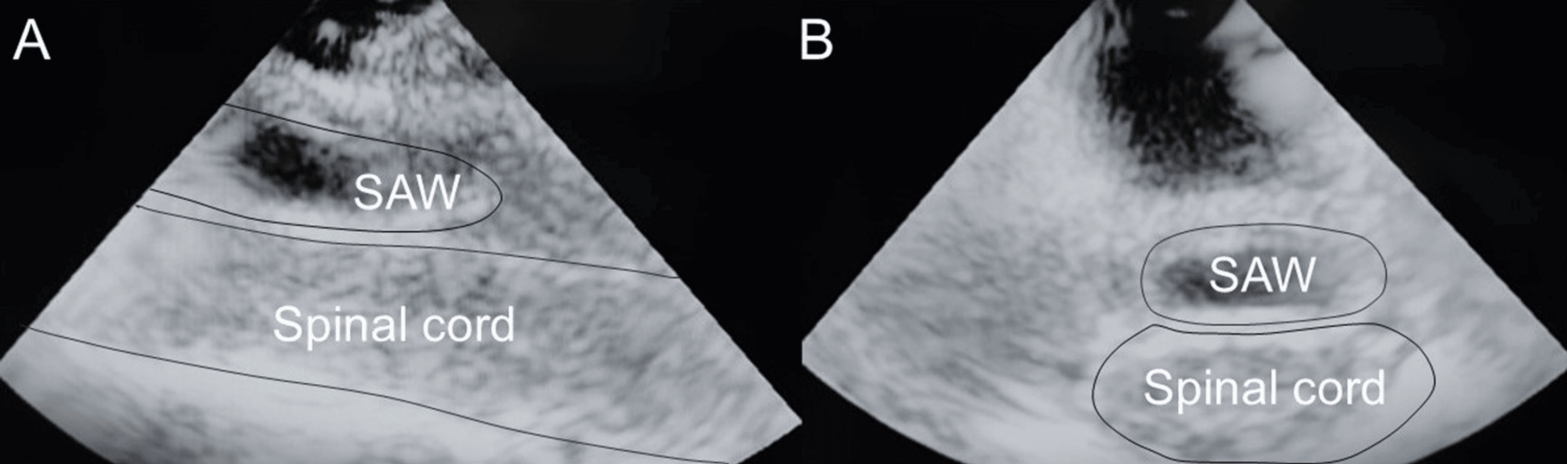
(A) Sagittal view and (B) axial view confirm spinal cord compression caused by SAW. SAW: spinal arachnoid web

**Figure 3:**
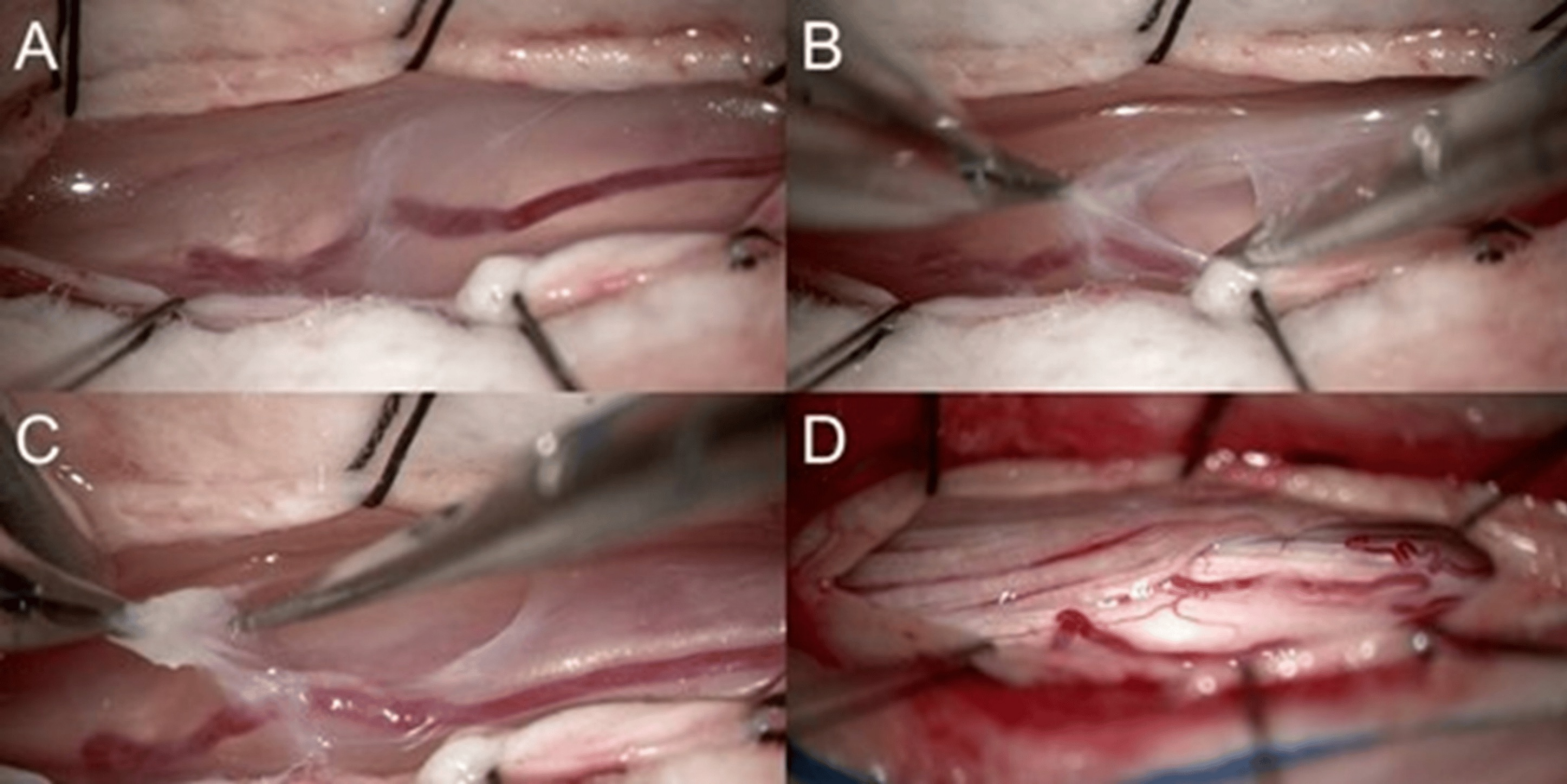
(A) The spinal cord is visibly compressed by a pressurized arachnoid web resulting from the obstruction of CSF flow. (B, C) The SAW is carefully resected using microsurgical techniques. (D) After resection, the spinal cord and nerve roots are released from compression. SAW: spinal arachnoid web; CSF: cerebrospinal fluid

Surgical intervention was performed one week after symptom onset. A laminectomy was performed from T3 to T5, during which intraoperative ultrasound imaging revealed a lesion moving synchronously with CSF pulsations (Figure 2). CSF trapped by the thickened arachnoid membrane was observed following the opening of the dura, exerting pressure on the spinal cord and displacing it ventrally. The developed membranes were meticulously resected using microsurgical techniques (Figure 3A-3C). After the complete removal of these tissues, intraoperative findings confirmed restored CSF flow and the resolution of spinal cord compression (Figure 3D).

**Figure 4:**
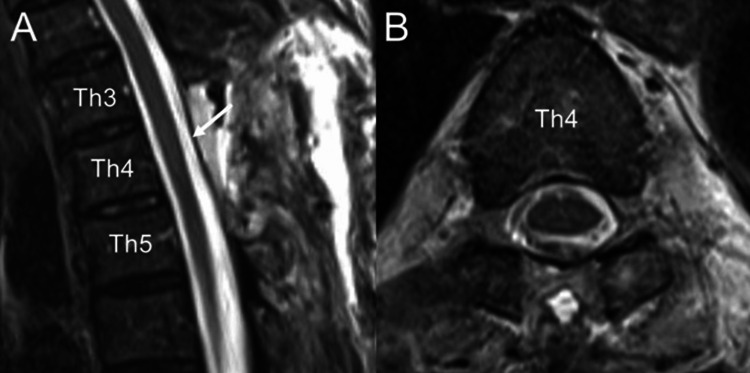
(A, B) Sagittal and axial T2-weighted images show the realignment of the spinal cord following the resection of the SAW (white arrow). SAW: spinal arachnoid web

A postoperative T2-weighted MRI performed one week later demonstrated the resolution of spinal cord distortion and its normalization (Figure 4A, 4B). Postoperative neurological improvement was significant. The patient achieved 5/5 strength in all lower extremity muscles on the Manual Muscle Test, compared to the preoperative 4/5 strength. Sensory function, including proprioception and vibratory sensation, was fully restored, and bladder and bowel function were completely regained, with no residual deficits. The patient was discharged in stable condition two weeks after surgery, with full resolution of his neurological symptoms. A written agreement for publication was obtained from the patient.

## Discussion

SAW, a rare and often underdiagnosed disorder with a chronic onset, is characterized by web-like structures and syringomyelia resulting from the obstruction of CSF flow [1]. Here, we report a surgical case with intraoperative findings consistent with typical features of SAW, despite the absence of common preoperative imaging and an acute clinical presentation. 

The scalpel sign, resembling the blade of a scalpel, is a characteristic imaging finding on sagittal MRI and CT myelography [8]. However, given the rarity of this entity, the frequency of positivity for its distinctive sign is unclear, and it would be also uncertain if it provides a definitive diagnostic pathway. In recent years, advanced imaging techniques, including heavily T2-weighted MRI directly visualizing the web-like structure and pinpointing its location and cine MRI demonstrating the pulsatile motion of the SAW with CSF flow, could be useful [9,10]. Given the invasive nature of both MRI and CT myelography, MRI remains a valuable tool; however, CT myelography is particularly useful for differentiating between SAW and spinal arachnoid cysts [11]. Proposed triggers involve congenital remnants such as a cephalic remnant of the septum posticum, trauma, inflammation, prior spinal surgery, subarachnoid hemorrhage, and degenerative processes, which result in the formation of a thickened arachnoid membrane that compresses the spinal cord or induces syringomyelia [1,2,12]. Although the characteristic sign was not observed in this case, the lesion's dorsal location in the thoracic spine, combined with the patient's history of subarachnoid hemorrhage and prior spinal surgery, aligns with the presentation of SAW. This mechanism is thought to begin with a decrease in CSF velocity caused by focal arachnopathy, leading to increased intralesional pressure that directly compresses the spinal cord [13]. Therefore, we believe that simple initial imaging findings indicating spinal cord deformation or displacement, as demonstrated in the present case, should be carefully considered in clinical practice, as they may represent an early stage of the pathophysiology.

Acute onset is an uncommon presentation of SAW, as most cases exhibit a chronic and slowly progressive course. Previous studies reported that approximately half of the patients experienced symptoms for over one year before surgery, with some studies noting durations as long as 4.6 years [2,14]. The last systematic review demonstrated an average duration of 38.9 months from symptom onset to treatment [15]. On the other hand, a case of SAW with acute progressive myelopathy within two weeks of onset was previously reported [16]. That case presented a symptomatic syrinx extending into the cervical spine requiring drainage, which differs from our case. Typically, sustained and long-term CSF occlusion results in the chronic development of myelopathy; however, the acute onset suggests that the transient nature of SAW, potentially acting as a check-valve mechanism or associated with inflammation, may have led to a sudden increase in pressure, resulting in rapid spinal cord compression, progression of injury, or ischemia.

Our case also demonstrated the effectiveness of surgical intervention. Microsurgical web excision with laminectomy or laminoplasty remains the standard surgical treatment for symptomatic SAW [2,7]. Intraoperative ultrasound, correlated with preoperative evaluation, facilitates surgical planning [17]. While the benefits of surgery in symptomatic cases are well-documented, cases with non-characteristic imaging findings like ours or minimal clinical symptoms that complicate diagnosis may not always warrant surgical intervention. Delayed intervention can lead to irreversible damage; however, the lack of evidence-based guidelines makes it challenging to determine the optimal timing for surgery in less severe cases [14,18,19]. This highlights the need for further studies to establish criteria for surgical intervention and to identify factors predictive of poor outcomes or non-response to treatment. Consequently, we emphasize the importance of timely intervention during the reversible phase of neurological symptoms, particularly in cases with atypical imaging findings, as observed in this instance. Additionally, even in cases of rapidly progressive neurological symptoms, it is crucial to consider SAW as a differential diagnosis and conduct a thorough examination to ensure accurate and timely management.

## Conclusions

The diagnostic challenge posed by SAW cases without characteristic preoperative findings underscores the importance of identifying spinal cord deformation or deviation during primary assessments and conducting prompt advanced examinations. Timely intervention to mitigate further deterioration may be required for acute progressive SAW.
